# Single‐Cell Profiling: Any Scale, Any Size, All at Once

**DOI:** 10.1002/advs.202518479

**Published:** 2025-11-28

**Authors:** Denise Goh, Felicia Wee, Rachel Elizabeth Ann Fincham, Ruisi Li, Joe Yeong

**Affiliations:** ^1^ Institute of Molecular and Cell Biology (IMCB) Agency for Science, Technology and Research (A*STAR) Singapore 138673 Singapore; ^2^ Department of Anatomical Pathology Singapore General Hospital Singapore 169856 Singapore; ^3^ Department of Microbiology Singapore General Hospital Singapore 169856 Singapore; ^4^ Duke‐NUS Medical School Singapore 169857 Singapore

**Keywords:** cellular heterogeneity, high‐throughput analysis, molecular profiling, single‐cell technology

## Abstract

Single‐cell technologies have revolutionized the understanding of cellular heterogeneity, revealing distinct cell populations and functional states with molecular precision. Traditional approaches, however, are constrained by cellular dimension and throughput scale, in addition to its inability preserve spatial information. Recent innovations now allow high throughput, large‐sized cell, and multi‐modal profiling of patient samples, overcoming these limitations. In this perspective, three recently developed single‐cell technologies is described, highlighting how their enhanced throughput, resolution, and sensitivity provide deeper insights into cellular identity and function.

## Graphical abstract

Traditional single‐cell technologies are constrained by cellular dimension and throughput scale, in addition to its inability preserve spatial information. With recent innovations, these technologies now allow high throughput, large‐sized cell, and multi‐modal profiling of patient samples, overcoming these limitations. Here, we describe three recently developed single‐cell technologies, highlighting how their enhanced throughput, resolution, and sensitivity provide deeper insights into cellular identity and function.

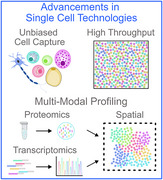

## 1 Main

Bulk RNA sequencing provides limited insights into the clinal composition of tumors and the tumor microenvironment, often masking cellular heterogeneity by averaging gene expression across cells.^[^ ^1^ ^]^ Overcoming this limitation, single‐cell RNA sequencing enables transcriptome profiling at single‐cell resolution, providing insights into tissue heterogeneity and facilitating the study of intricate molecular and genetic mechanisms.^[^ ^2^ ^]^ However, these approaches are often constrained by cellular dimension and throughput scale, which typically profiles only up to ≈30 000 cells per run.^[^ ^3^ ^]^ New technologies now enable single‐cell profiling of large multinucleated cells, along with the ability to analyze millions of cells per experiment, offering a scalable, high‐throughout, and cost‐effective solution to traditional approaches. When combined with multi‐modal capabilities, these technologies can capture diverse molecular layers to provide a comprehensive view of cellular identity and function. Below, we describe and highlight the potential of three cutting‐edge single‐cell technologies in overcoming traditional limitations (**Table** **1**).

**Table 1:** Comparison of three single‐cell technologies highlighting their key differences and applications.

Parameter	Stereo‐cell	STAMP	ICOS
Approach	RNA capture using DNA nano‐ball technology	Imaging‐based single‐cell profiling on‐slide, using commercial staining panels	Imaging‐based single‐cell profiling on‐slide, using commercially available antibodies
Type of modality	RNA and protein/epitope	RNA, protein, morphology	Protein, morphology
Sample compatibility	Cell suspension (i.e., PBMCs, cell lines, extracellular vesicles, oocytes, large cell types, protists)	Cell suspension (i.e., PBMCs, stem cells, cancer cells, cell lines, cancer associated fibroblasts) Nuclei suspension (extracted from fresh or FFPE tissues)	Cell suspension (i.e., PBMCs, buffy coat, cell lines, cytopathological fluids)
Throughput	Up to 900 000 cells	100–5 000 000 cells	Up to 300 000 cells per slide
Sequencing depth	>20 000 reads per cell (PBMCs)	Up to 6000 genes	Not applicable
Number of Protein Biomarkers	mIF: 4 (including DAPI and wheat germ agglutinin) Stereo‐cell CITE: 154 (epitope profiling)	Up to 64	7 (including DAPI)
Cost	Not Available	$0.003 per cell	Not Available
Applications demonstrated	Cell profiling and phenotyping (immune and non‐immune)	Cell profiling and phenotyping (immune and non‐immune)	Cell profiling and phenotyping (immune and non‐immune)
	Detection of rare cell types	Detection of rare cell types	
	Cell‐cell interaction and cell migration studies	Immune cell perturbation experiments,	Identification of cell therapy products
	Capture and sequencing of large microstructures	Profiling of dissociated tissues	
	Capture of extracellular transcriptomes		
Distinct strengths	Unbiased cell capture, including large cell types	Facilitates detection of rare cell types	Long term storage (−20 °C) of unstained slides
	Facilitates integration with imaging‐based modalities and multi‐omics workflows	Non‐destructive, sequencing‐free single cell genomic technique	Potential to be harnessed as a quality control tool in the manufacturing of cell therapy products
	Supports cell culture	Cost‐Effective	Enables AI model development
Limitations	Complex chip fabrication and data analysis	Unsuitable for full length transcript analysis	Multiplexing capability limited to seven biomarkers in a panel
	Susceptible to lateral RNA diffusion (low‐level transcript contamination)	Relies on predefined gene panels	Does not capture sequencing data
	Lack of imaging protocols for larger chips	Probe design constraints make mutation and immune receptor profiling challenging	

This table summarizes the key characteristics of three recently developed single‐cell platforms, which are promising alternatives to conventional single‐cell sequencing approaches that are constrained by cellular dimension and throughput scale.

Abbreviations: artificial intelligence, AI; formalin‐fixed, paraffin‐embedded, FFPE; peripheral blood mononuclear cell, PBMC.

### 1.1 Stereo‐Cell Technology: High‐throughput Multi‐Modal Single‐Cell Profiling

With sequencing patented DNA nanoball technology and in situ RNA capture technology, the SpaTial Enhanced Resolution Omics‐sequencing (Stereo‐seq) platform was developed to offer unbiased whole‐transcriptomic profiling at near‐cellular resolution.^[^ ^4^ ^]^ Although it has shown promise in supporting transcriptomic analysis of well‐structured healthy tissues,^[^ ^5^ ^]^ its performance substantially reduced in pathological tissues with high structural complexity and heterogeneity.^[^ ^4^, ^6^ ^]^ As a result, it often necessitates a trade‐off in spatial resolution to maintain sufficient statistical power. This challenge highlights the distinct advantage of the subsequent SpaTial Enhanced Resolution single‐cell sequencing (Stereo‐cell) platform,^[^ ^7^ ^]^ which also leverages on the inherent superiority of the DNA nanoball chip to enable scalable, unbiased, and high‐resolution single‐cell profiling.

#### 1.1.1 High‐Throughput and Robust Performance

It accommodates a wide range of input sizes, from 200 to nearly one million cells per chip. With deep learning‐based cell segmentation and imaging‐based doublet elimination, Stereo‐cell analysis of peripheral blood mononuclear cells (PBMCs) demonstrated superior capture efficiency and specificity compared to mainstream technologies, such as Chromium, Drop‐seq, and Smart‐seq2. This robust performance enabled accurate cell profiling and phenotyping, producing results that closely resemble those obtained by flow cytometry. Its ultra‐high‐throughput capability (up to 900 000 PBMCs) facilitated more detailed classification of cell populations–compared to profiling at lesser cell quantities–and the detection of rare cell types, including hematopoietic stem and progenitor cells.

#### 1.1.2 Compatibility with Large and Complex Cell types

Stereo‐cell was applied to profile a wide range of cell populations, including PBMCs, cell lines (e.g., NIH‐3T3 and HEK293T), large‐sized microstructures (e.g., multinucleated skeletal myofibers), oocytes (the largest cell type in mammals), and extracellular vesicles. Due to size and structural complexity, large and complex cell types are often not compatible with microfluidic‐based methods and thus, studied at low‐throughput. Showcasing its unbiased cell‐size capture capability, Stereo‐cell effectively captured and sequenced multinucleated skeletal myofibers and oocytes. Beyond standard PBMCs, Stereo‐cell preserved the spatial position of individual nuclei and robustly identified spatial segments within the skeletal myofibers. It also efficiently characterized mouse oocytes by detecting genes related to maturation, chromatin remodelling, and subcellular gene expression localization.

With these observations, Stereo‐cell demonstrated its potential to profile other large cell types, such as hepatocytes, cardiomyocytes, and adipocytes, which are typically a challenge for conventional single‐cell platforms.

#### 1.1.3 Multi‐Modal Integration and Potential Microbial Applications

The Stereo‐cell platform is compatible with imaging‐based modalities (e.g., multiplex immunohistochemistry) and multi‐omics approaches (e.g., CITE‐seq) to profile transcriptome and protein/epitope simultaneously at single‐cell resolution. This capability can provide additional insights by uncovering subtle differences in cellular characteristics under varying conditions that remain imperceptible through transcriptomic analysis alone.^[^ ^8^, ^9^ ^]^ With high spatial resolution, Stereo‐cell also supports cell culture, thus allowing direct assessment for changes in gene expression levels driven by cell‐to‐cell contact and cell migration while avoiding alterations caused by cell dissociation. Using cultured fibroblasts as a paradigm, comparison between in situ capture of transcriptome and dissociated cells revealed that the former had high gene expression related to extracellular matrix organization, cell migration, and cell‐substrate adhesion while the latter had high expression of apoptotic genes. Further analysis subsequently revealed 11 cell states, including distinct cell cycle phases and transcriptional profiles, and captured fibroblast response to lipopolysaccharide stimulation. Having demonstrated its success in profiling cultured cells, Stereo‐cell has the potential to extend beyond mammalian systems to support microbiota, fungi, parasites, and plant research—provided it offers flexible environmental controls and can be adapted to the specific needs of each organism type. Such applications could open new avenues for studying host–microorganism interactions, pathogen dynamics, and plant‐specific developmental or stress‐response processes at single‐cell resolution.

### 1.2 STAMP Technology: Sequencing‐free Platform for Multi‐Sample Single‐cell Profiling

Single‐cell Transcriptomics Analysis and Multimodal Profiling (STAMP) is an imaging‐based technique that offers single‐cell profiling on‐slide.^[^ ^10^ ^]^ A key distinction from Stereo‐cell is that STAMP captures and preserves cells at a fixed point in time, locking in their shapes and expressed genes, rather than allowing the assessment of dynamic changes over time.

#### 1.2.1 Sequencing‐Free, Cost‐Effective, and Efficient Workflow with Broad Platform Compatibility

Single‐cell transcriptomics is costly and requires extensive sequencing to extract useful information from each cell. Overcoming this challenge, the STAMP workflow immobilizes cell suspension onto glass slides to form a monolayer for sequencing‐free single‐cell analysis of various cell types, through imaging platforms. This approach significantly brings down the cost from $0.10 to $0.003 per cell as compared to conventional techniques. Furthermore, its compatibility with a range of commercial imaging platforms, RNA and/or protein panels, experiment designs, and sample types, reinforces STAMP as a scalable, and flexible solution single‐cell profiling.

#### 1.2.2 Simultaneous Profiling of Multiple Samples and Diverse Cell Types

Pitino et al.,^[^ ^10^ ^]^ demonstrated the capabilities of STAMP to perform sequencing‐free single‐cell genomics, even at low‐input cell numbers. In a multi‐sample configuration with PBMCs, stem cells, cancer‐associated fibroblasts, and cancer cell lines, STAMP enabled simultaneous analysis of the various cell types across different imaging platforms (i.e., Xenium, CosMx, MERSCOPE, and Phenocycler Fusion). Given the imaging‐based nature of STAMP, having the cells fixed on a microscopic slide could enable profiling of irregularly‐shaped cells, such as neurons, overcomes the limitations of microfluidic‐based methods which are better suited for spherical or simpler cell shapes.

#### 1.2.3 High‐Resolution Immunophenotyping and Rare Cell Detection

Cells are analyzed using gene‐ or protein‐specific probe or antibody panels to capture both targeted measurements and whole transcriptome analysis. High resolution immuno‐phenotyping of PBMCs yielded equal or superior findings compared to single‐cell assays at detecting lineage marker genes–it generated a map of 31 immune cell states, paving the way for large‐scale atlas‐building projects. Additionally, STAMP also successfully detected rare cell types which accounted for 0.001% of the overall sample size, demonstrating its impressive sensitivity and potential in clinical application.

Due to the non‐destructive nature of STAMP, samples can be analyzed across different instruments and subsequent spatial proteomic profiling can be performed to generate a multi‐omics dataset of transcript and protein measurements. Moreover, since cells were immobilized and intact, this STAMP can also extract morphological data from hematoxylin and eosin (H&E) staining to enable combined analysis of molecular states and tissue architecture. These capabilities showcase the robustness of the technology. Its sequencing‐free and scalable design also renders it particularly suitable for phenotyping complex microbial communities, where low‐cost, high‐throughput approaches are critical to capture community structure and rare microbial subpopulations.

### 1.3 ICOS Technology: High‐throughput Phenotyping Workflow for Fluid‐derived Samples

Auto‐Imaging Cytometry On the Slide (ICOS) is developed for phenotyping cells suspended in fluids, including samples such as cell lines, cytopathological fluids, blood, and chimeric antigen receptor (CAR) T cell products, with the use of antibodies for spatial protein detection.^[^ ^11^, ^12^ ^]^

#### 1.3.1 Automated Workflow for Fluid Samples with Efficient Operation and Long‐term Morphology Preservation

Similar to STAMP, ICOS involves spreading the cells as a monolayer on positively‐charged glass slides, which are compatible with autostainers for automated multiplex immunohistochemistry staining following cell fixation, and digital pathology scanners for automated fluorescent image acquisition. This efficient workflow is capable of handling high volumes of slides and requires less manual intervention compared to traditional methods that are time consuming, labour‐intensive, and have higher inter/intra‐batch variability. Moreover, similar to how cells suspended in fluids are typically stored, ICOS slides can also be stored at −20 °C long term to preserve cellular morphologies after smearing, until ready for staining, facilitating parallel analysis of longitudinal samples.

#### 1.3.2 Downstream Spatial Analysis and AI‐driven Prediction

The acquired high‐resolution multiplexed images from ICOS slides can be piped into digital pathology analytics software to conduct downstream qualitative and quantitative single‐cell analysis, thus facilitating high‐throughput cell phenotyping using fluorescence signal intensity. Additionally, using CD3 as a paradigm, Wee et al. further developed an AI‐driven prediction model that can differentiate CD3^+^ and CD3^−^ cells on unstained greyscale images from ICOS cell smears.^[^ ^12^ ^]^ By leveraging learned features of cellular morphology, the AI model assists in identifying T cells from unlabelled greyscale images before immunohistochemical staining is completed. The predicted T cell counts can then be tabulated for each sample to screen for patients with abnormally low or high T cell levels. This concept can be extended to other protein biomarkers to develop similar screening applications tailored to specific clinical or research use cases.

#### 1.3.3 Cross‐Modality Integration with Current Limitations in Multiplexing Capability

The ICOS pipeline also supports cross‐modality studies whereby the same slides that were subjected to multiplex immunohistochemistry can also be stained with H&E (and potentially other histological stains). This allows the integration of various approaches for further evaluation on the same cells. However, ICOS faces limitation in multiplexing capability. Currently the maximum tested number of markers in an ICOS panel is seven, inclusive of DAPI, thereby limiting the depth of immunophenotyping. Further tests are necessary to assess if deeper molecular profiling can be conducted on ICOS slides.

### 1.4 Integration Trends and Future Perspectives of Multi‐Modal Single‐Cell Technologies

Single‐cell technologies have fundamentally reshaped our ability to explore cellular complexity by integrating transcriptomic, proteomic, and other modalities in both tissue and suspension contexts. These advances—from early RNA sequencing to cutting‐edge platforms—have illuminated the cellular heterogeneity and microenvironmental interactions that drive disease development and progression, along with therapeutic response. Complementing these technologies with robust computational tools that can accurately segment cells and integrate diverse molecular modalities is equally important, and will further reveal biologically meaningful spatial patterns that unlocks unprecedented insights into tissue organization and cellular function. However, to translate these technologies into clinical settings will require an expansion of multiplexing capabilities and standardizing workflows.

Building on the complementary capabilities of Stereo‐cell, STAMP, and ICOS, the key priority is not merely adding more readouts, but integrating heterogeneous measurements into robust, clinically interpretable representations. First, cross‐modality alignment remains fragile across platforms with differing capture chemistries, dynamic ranges, and spatial resolutions, especially for large or multinucleated cells (Stereo‐cell), multi‐instrument imaging panels (STAMP), and slide‐based protein readouts with limited plex (ICOS). We advocate physically anchored co‐assays (shared barcodes, fiducials or standardized co‐stains) and routine internal controls/spike‐ins to bound alignment error and quantify modality‐specific bias. Second, at scale, pipelines must handle batch effects, missing modalities, and spatial autocorrelation while preserving biology (lineage/clonotype structure, neighborhood topology). This calls for interoperable data formats, scalable latent‐space models with calibrated uncertainty for cross‐modal prediction, and task‐defined benchmarks that score biological fidelity (e.g., recovery of known spatial gradients or clonal phylogeny) rather than aggregate error alone. Third, clinical translation depends on pre‐analytical standardization (sampling, fixation), turnaround time and cost, and explainability. A practical route is a two‐tier strategy: use high‐plex discovery (e.g., Stereo‐cell or imaging panels) to learn minimal disease signatures and spatial contexts, followed by validated low‐plex panels and lightweight surrogate models (including ICOS‐style workflows) for routine use with clear quality control gates, drift monitoring, and decision thresholds. Integrating multi‐omics technologies with computational tools will unlock unprecedented insights into tissue organization and cellular function, driving advances in precision medicine and our fundamental understanding of biology.

These approaches may also ultimately bridge mammalian, plant, and microbial systems, enabling a holistic understanding of host–microbiota interactions, pathogen dynamics, and ecological complexity at single‐cell resolution, albeit a different set of challenges. For instance, extending these frameworks to host–microbiota contexts will additionally require contamination‐aware pipelines and cross‐kingdom references. Overall, progress will be fastest where chemistry and computation are co‐designed, community standards converge, and evaluation prioritizes biological consistency and clinical actionability over breadth alone.

## Conflict of Interest

The authors declare that they have no conflict of interest.
